# The independent legal entity reform of emergency medical centers in China: opportunities and challenges

**DOI:** 10.1038/s44401-025-00065-w

**Published:** 2026-01-28

**Authors:** Quan Wang, Yumeng Lv, Yixin Qin, Yingming Song, Aifeng Ren, Yang Zheng, Hailing Yu, Li Yang

**Affiliations:** 1 School of Public Health, Peking University, Beijing, China; 2 Saw Swee Hock School of Public Health, National University of Singapore, Singapore, Singapore; 3 Jinan Medical Emergency Center, Jinan, Shandong Province China; 4 Beijing Center for Public Health Emergency Management, Beijing, China; 5 Beijing Institute for Health Development, Peking University, Beijing, China; 6 National Health Commission Key Laboratory of Health System Reform and Governance (Peking University), Beijing, China

**Keywords:** Health care, Health humanities, Medical humanities, Social policy

## Abstract

Beijing’s district-level reform of emergency medical centers represents a pivotal step in strengthening the city’s prehospital emergency medical system. The reform aims not only to enhance workforce stability and financial transparency but also to improve system-wide management and coordination across municipal, district, and local levels. While these efforts have improved efficiency and accountability, challenges persist, including limited funding, uneven capacity, and unclear career pathways for non-technical staff. Beijing’s experience highlights the need for differentiated, context-specific strategies to balance autonomy and integration, ensuring the sustainable, equitable, and safe development of China’s prehospital emergency services.

## Introduction

By the end of July 2024, Beijing has completed the administrative approval process to establish all district-level emergency medical centers as independent legal entities^1^, marking a significant milestone in China’s ongoing reform of prehospital emergency medical services. By mid-2025, all of district-level emergency medical centers had completed the necessary administrative procedures and begun operating as independent legal entities. Beijing’s approach offers a valuable model for other regions in China. According to the *China Health Statistical Yearbook*, there were 345 major emergency centers in 2015, which increased to 526 by 2021^2^, reflecting a 52.5% growth over six years. This expansion demonstrates China’s ongoing commitment to strengthening the accessibility and responsiveness of prehospital care. While the reform aims to improve the management and service quality of emergency medical services, it also presents new challenges for ensuring long-term sustainability and maintaining high safety standards in service delivery. We analyze the advantages and disadvantages of the independent legal entity reform and examines its broader implications for other regions in China, along with considerations for future reforms.

## The prevail of affiliation model

Prehospital emergency medical services (EMS) are a crucial component of public safety and healthcare systems. Prehospital EMS refers to the provision of emergency medical care outside the hospital setting, including the receipt of emergency calls, dispatch of ambulances, on-site triage, and stabilization and transport of patients to appropriate medical facilities. In contrast, in-hospital EMS encompasses the diagnosis, resuscitation, and definitive treatment provided after patients arrive at the hospital. The two systems are inherently interdependent: the prehospital EMS provides the first link in the “chain of survival,” while in-hospital EMS continues the continuum of care necessary to improve patient outcomes^3^. As urbanization in China accelerates, population density, traffic congestion, and frequent public health emergencies have led to an increased demand for these services. Given this growing demand, improving the efficiency, service quality, and financial transparency of prehospital EMS has become a key priority for public health policy. High-quality, well-coordinated, and safe emergency medical services are essential to improve patient outcomes and optimize system performance (BOX 1).

Prehospital and in-hospital emergency medical services should be seamlessly integrated, as this is a crucial factor in reducing system delays and improving patient health outcomes^4^. However, in China, these services are typically handled by two separate departments in system design: emergency medical centers/stations manage prehospital care, while hospital emergency departments handle in-hospital care. In practice, emergency medical centers/stations can function as independent departments, operating as legal entities with their own medical staff and ambulance teams. Alternatively, majority of emergency medical centers/stations exist as departments within hospitals or primary care institutions, directly managed and operated by the affiliated institution^5,6^.

In recent decades, the rationale for the affiliation model has become evident. Due to insufficient financial support from the government, health insurance reimbursements typically do not cover the full costs of medical staff and ambulance services. For example, in Beijing, government subsidies for prehospital care per call amount to RMB 650 (about 90 USD), far less than the actual costs. Under the affiliation model, these deficits can be offset by other clinical departments within the hospital. Therefore, the affiliation model remains the most common structure across China, particularly in less-developed areas where government financial resources are limited^7^. Moreover, the lack of a medical priority dispatch system and a comprehensive information-sharing channel between hospitals has highlighted another advantage of the affiliation model: it fosters closer coordination between prehospital teams and hospital emergency departments, thereby reducing delays in patient care, which increased the integration and quality of service.

However, the disadvantages of the affiliation model have become increasingly apparent. While it strengthens the connection between prehospital and in-hospital emergency services, it weakens the relationship between emergency medical centers/stations and the emergency command department. Prehospital teams tend to transport patients to their affiliated institutions, even when these may not be the most appropriate facilities. Additionally, the absence of an integrated information-sharing system between hospitals can result in patients being taken to hospitals ill-equipped to handle their conditions, necessitating further transfers and causing delays^8,9^. Smaller institutions, such as primary care centers and private hospitals, often lack the resources to maintain a full-time prehospital medical team. In these cases, staff are temporarily recruited from other clinical departments to serve as prehospital personnel for short periods. Such ad-hoc teams are highly unstable and experience frequent turnover, making it difficult for physicians to accumulate sufficient prehospital emergency experience. As a result, these teams often lack the necessary expertise and operational efficiency, leading to lower quality of care, reduced patient safety, and overall weaker performance^10^.

A critical disadvantage of the affiliation model is its fragmented nature, which weakens the integration between EMS and the public health system. In some areas of China, the link between hospitals and public health institutions, such as the Centers for Disease Control and Prevention (CDC) and health education organizations, is underdeveloped. Although emergency medical centers/stations are officially recognized as public health institutions under the *Primary Health Care, Medicine and Health Promotion Law*, the affiliation model limits their ability to collaborate effectively with public health agencies. For instance, the early administration of CPR significantly improves patient survival rates, but the lack of coordinated training and public access to AEDs illustrates the inefficiencies of the affiliation model. The implementation rate for bystander CPR in China is low (4.5% in 8 large and medium-sized cities around China), which is much lower than it in Canada, Europe, or the US^11,12^.

Box 1 Four key functions of the prehospital EMS system in China **Function 1: Emergency medical response and patient transport**

The core function of the prehospital EMS system is to provide timely emergency response and patient transport. Members of the public can call the emergency hotline (typically *120* in China) to request an ambulance. Upon receiving the call, the command center dispatches an appropriate team to the scene, provides on-site assessment and stabilization, and transfers the patient to a suitable medical facility for further treatment. This function represents the daily operational foundation of the prehospital EMS system and is critical for reducing avoidable delays in emergency care.

**Function 2: On-site medical support for major events**

Prehospital EMS systems play an important role in ensuring medical preparedness and safety during large-scale public events—such as sports competitions, concerts, festivals, exhibitions, celebrations, and high-level meetings. Local health authorities or government departments typically deploy prehospital EMS teams to provide on-site ambulance coverage and medical standby throughout the event. This arrangement enables rapid medical response in the event of sudden illness or injury, ensuring the safety of participants and the public.

**Function 3: Response to public emergencies and disasters**

During major emergencies—such as natural disasters, large-scale traffic accidents, fires, or public health emergencies—prehospital EMS teams are mobilized under the unified command of local authorities to participate in emergency rescue operations. Depending on the nature of the event, prehospital EMS teams often work collaboratively with other departments, including the fire brigade, police, and civil defense. This function reflects the EMS system’s critical role in disaster response and its integration within the broader public safety framework.

**Function 4: Public health education and first aid training**

Beyond emergency response, prehospital EMS agencies also contribute to public health education by promoting emergency preparedness and first aid knowledge among the general population. This includes training citizens in basic life support skills such as cardiopulmonary resuscitation (CPR), the use of automated external defibrillators (AEDs), and the initial management of common medical emergencies. These community-oriented activities not only improve public awareness but also enhance population-level resilience to sudden health crises.

## Historical evolution of Beijing’s prehospital emergency medical service system

**Fig. 1:**
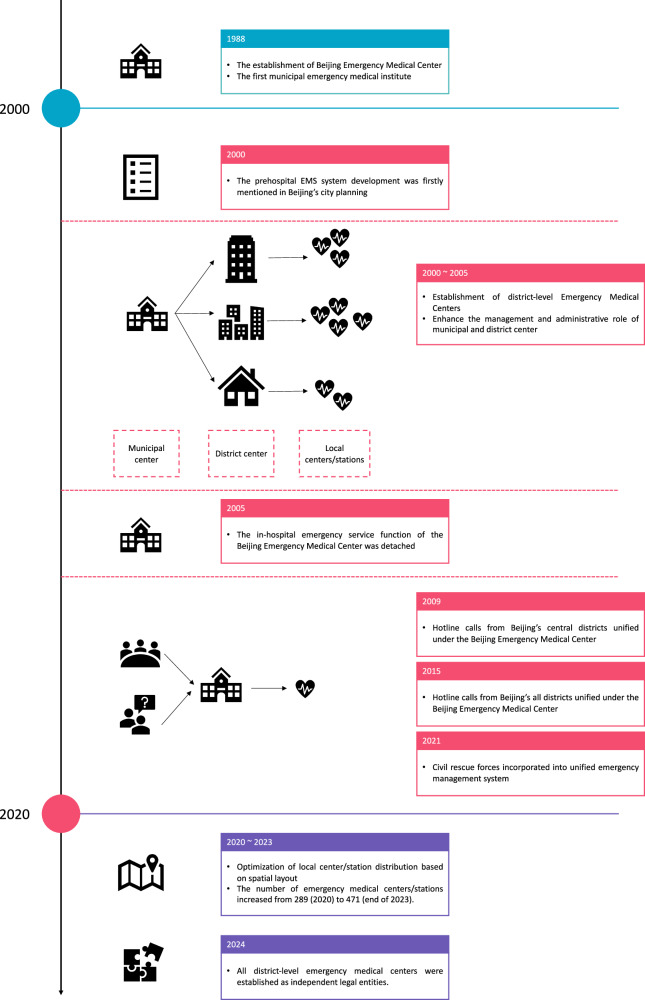
The evolution of Beijing prehospital EMS system.

No reform takes place on a blank slate; Beijing’s recent transformation toward independent legal entity emergency centers is deeply rooted in the institutional and operational evolution of its prehospital emergency medical service system over the past three decades (Fig. 1).

Beijing established the Beijing Emergency Medical Center (BEMC) in 1988, marking the creation of the city’s first municipal-level emergency medical center. At its inception, the BEMC functioned not only as a prehospital emergency service provider but also as an emergency hospital. It operated clinical departments such as trauma surgery and acute cardiovascular and cerebrovascular care, and offered inpatient services.

In 2000, prehospital emergency medical services were formally incorporated into Beijing’s urban development plan^13^. Over the following five years, district-level emergency medical centers were gradually established across the city’s administrative districts. During this period, both municipal and district-level centers saw their administrative and managerial roles strengthened. Beyond serving as technical units responsible for ambulance dispatch and prehospital medical services, they also began to assume management responsibilities for local centers/stations within their jurisdictions. The establishment of district-level centers was driven by the need to address Beijing’s large geographic area and the transportation challenges in its remote mountainous districts. These local centers improved the city’s capacity to respond promptly to emergencies and manage prehospital resources in line with local conditions. To align with this evolving policy orientation, the BEMC subsequently separated its in-hospital emergency and inpatient service functions, dedicating itself exclusively to prehospital emergency care^14^.

Before 2009, the absence of an integrated information and communication system meant that emergency patients typically contacted the nearest hospital directly, as there was no unified call-handling mechanism. In 2009, Beijing centralized emergency call handling for the core urban districts under the BEMC, and by 2015, all remaining districts had been integrated into this system. Under the unified command of the BEMC, incoming emergency calls were assessed and directed to the most appropriate local center/station, which then dispatched an ambulance team to the scene (BOX 2).

In 2020, Beijing released its first spatial planning for emergency medical system, which guided the optimization of resource distribution and service coverage^15^. Over the subsequent three years, the number of emergency medical centers/stations expanded rapidly, reflecting the city’s ongoing commitment to strengthening its prehospital emergency system.

In summary, the development of Beijing’s prehospital EMS system—from a hospital-affiliated model to a citywide integrated network—laid the organizational and institutional foundation for the later reform toward independent legal entity emergency centers, which aimed to further enhance professionalism, coordination, and service efficiency.

Box 2 Prehospital emergency command centers Prehospital Emergency Command Centers are a key component of China’s emergency medical service (EMS) system. Their primary function is to receive emergency calls, assess the situation, and dispatch the most appropriate prehospital emergency response team. The organizational structure of command centers varies across cities depending on local governance, resource availability, and administrative arrangements.

In large metropolitan areas, command centers are typically established as independent institutions with substantial autonomy in finance, personnel management, and recruitment. They are responsible for coordinating and allocating resources across all emergency medical centers/stations within the city, ensuring efficient task distribution, timely response, and optimal use of emergency resources.

In contrast, in some cities, especially those small and medium-sized cities, where financial and human resources are more constrained, command centers are often affiliated with a major municipal hospital. In such cases, their personnel, budgeting, and management are integrated into the hospital’s administrative system. Notably, in these settings, emergency medical centers/stations are also commonly organized under the affiliation model. As a result, the prehospital emergency command center and the hospital-affiliated emergency medical center are frequently managed as a single clinical department—typically referred to as the Prehospital Emergency Department. This department operates both the ambulance teams and the call-handling staff responsible for receiving and managing emergency calls.

In some cities, the command center is further integrated into a comprehensive municipal emergency management center, which also includes departments such as fire, traffic, and public security. When a citizen calls the unified emergency hotline, the dispatcher coordinates the response and assigns the task to the relevant department, ensuring an integrated and multisectoral approach to emergency management.

In Beijing, the functions of the command center are carried out by the Beijing Emergency Medical Center, an independent institution with considerable autonomy in finance, personnel management, and recruitment. This center not only receives all medical emergency calls across the city and dispatches appropriate ambulance teams, but also operates its own high-quality prehospital medical service teams. In addition, it plays a crucial role in providing on-site medical support for major public events and in responding to large-scale emergencies and disasters.

## The independent legal entity model reform and policy drivers behind

The primary advantage of the independent legal entity model lies in the stability of both personnel and finances. Under this structure, emergency medical centers/stations are no longer affiliated with other medical institutions, allowing the prehospital service team to operate as a full-time, fixed unit. This ensures greater stability and consistency within the team, enhancing both the efficiency and quality of prehospital emergency services. Since 2020, the performance of the prehospital emergency system has continuously improved, accompanied by an increasing number of emergency medical centers and stations (Table 1).

**Table 1:** Key performance indicators of prehospital EMS system

	2020	2021	2022	2023	2024
Annual prehospital emergency dispatches (thousand cases)	709	871	920	891	895
On-site critical and severe cases (thousand cases)	483	599	/	708	690
Emergency call fulfillment rate (%)	96.9	96.6	99.5	99.9	100.0
Average emergency response time (minutes)	~18	~16	16.04	12.20	10.04
Number of emergency medical centers/stations	289	/	470	471	473

/: no publicly available data.

Data Source: Beijing Municipal Health Commission Statistical Reports 2021–2025.

Additionally, under the independent legal entity model, emergency medical centers/stations have direct control over their finances, promoting financial transparency and autonomy. This independence enables them to manage resources more effectively, allocate funds based on actual needs, and invest in critical emergency equipment or hire staff as required. Although the model may face short-term financial challenges, such as inadequate government subsidies, its enhanced financial autonomy is expected to support the long-term sustainable development of emergency centers.

However, the improvements in service quality and operational capacity were not the primary motivations for this recent reform. The main policy rationale lay in strengthening the internal management structure of the prehospital EMS system.

As discussed earlier, the establishment of district-level emergency medical centers between 2000 and 2005 aimed to enable each district center to coordinate and oversee the local centers/stations within its jurisdiction. Yet, prior to this reform, a substantial number of district-level emergency medical centers were affiliated with district-level public hospitals. In these cases, the director of the emergency center often concurrently served as the hospital’s emergency department head, medical affairs director, or even vice president. Under China’s current health system design, however, hospitals do not have administrative authority over other medical institutions within their geographic district. This structural arrangement meant that under the affiliation model, district-level emergency medical centers had no direct management channel to supervise or coordinate local centers/stations in their jurisdiction.

Consequently, district-level emergency medical centers could provide only limited professional guidance but were unable to systematically evaluate or manage the performance and quality of local centers/stations. Moreover, due to China’s hierarchical governance and tiered fiscal structure, government subsidies were typically allocated directly to the hospitals to which district-level emergency medical centers were affiliated. Because hospitals have no administrative authority over other institutions in their area, these funds lacked a transparent mechanism for redistribution to the local centers/stations that actually required support.

To fulfill these responsibilities, district-level emergency medical centers must be capable of assuming on-site command functions during emergencies—acting as local representatives of the Beijing Emergency Medical Center (BEMC) and coordinating directly with district government agencies to manage on-site rescue operations and patient transfers. It is worth noting that both academic and policy communities have previously debated whether all local centers/stations across Beijing should be placed directly under the administration of the BEMC, thereby abolishing district-level emergency medical centers to achieve greater system efficiency and flatten organizational hierarchies. However, such a structure would make it difficult for municipal personnel to reach remote districts quickly in the event of a disaster, while local centers/stations would lack the capacity to conduct on-site command and coordination.

Therefore, establishing district-level emergency medical centers as independent legal entities allows for stronger management and coordination within districts, bridging the operational and administrative gaps between the municipal-level command and the grassroots local centers/stations. Since 2020, the number of local centers/stations in Beijing has expanded rapidly—from 289 in 2020 to 473 by the end of 2024—making effective district-level oversight and coordination increasingly necessary. These newly developed local centers/stations have undergone standardized construction, with unified planning, spatial layout, facility standards, and staffing requirements, forming a coherent and well-structured emergency service network across the city. The reform responds to this growth by enhancing accountability, clarifying governance structures, and strengthening the district-level linkages essential for an efficient and resilient prehospital EMS system (BOX 3).

Obviously, the reform also faces several significant challenges. First, government subsidies for emergency medical centers/stations are currently insufficient to fully cover the high operational costs of emergency service dispatch^16^. This financial strain becomes particularly acute as emergency demand rises. Currently, the fee structure for emergency services is not well-established, and many services struggle to generate sufficient revenue.

Another critical issue is managing aging emergency personnel. Emergency work is physically demanding, and as staff age, they may find it difficult to perform the strenuous tasks required. In independent emergency centers/stations, the fixed positions offer limited opportunities for older staff to transition to less demanding positions. Unlike the affiliation model, where older staff can be reassigned to administrative or other clinical departments, independent centers/stations lack this flexibility. This highlights a limitation in personnel management under the independent legal entity model. Moreover, independent centers often struggle to recruit qualified doctors due to limited promotion opportunities and relatively low pay^17,18^, which further impacts the quality of care delivered. Another challenge of the independent legal entity model concerns the career development pathways for non-technical staff, such as dispatchers. Under the affiliation model, these employees followed a career trajectory similar to that of administrative staff within hospitals, supported by a well-established professional title and promotion system. However, under the independent legal entity model, such non-technical personnel lack a clearly defined professional title system and have no access to the civil service career track. This institutional gap limits their opportunities for career advancement and may undermine long-term workforce motivation and retention.

A further challenge with the independent legal entity model is in training and continuous learning. Due to their independence, prehospital teams typically only interact with patients for brief periods, limiting their ability to learn from patient outcomes. In contrast, prehospital teams under the affiliation model are involved in subsequent stages of treatment, allowing them to gain valuable insights and professional growth. This lack of learning opportunities for independent teams may hinder their professional growth and impede the overall development of prehospital medical expertise, ultimately impacting the quality of care provided.

To better illustrate this inherent tension and the corresponding policy responses, Table 2 presents a trade-off framework comparing the two models and identifying complementary policy mechanisms that can mitigate their respective risks.

**Table 2:** Trade-offs between integration and independence in prehospital EMS governance

	Affiliation model	Legal entity model	Complementary policy mechanisms
Coordination	Strong linkage between hospitals and EMS; seamless referral and communication	Risk of fragmentation across centers and weakened hospital coordination	Development of unified information platforms and shared dispatch systems
Professionalism	Staff rotation increases clinical exposure and hospital experience	Stable full-time EMS teams improve specialization	Joint training and continuing professional development programs
Financial management	Shared hospital budgets provide partial cross-subsidization	Transparent budgeting and independent financial control, but limited resources	Performance-based subsidies and fiscal earmarks for EMS operations
Accountability	Oversight embedded within hospital governance	Clear managerial accountability and defined administrative boundaries	Enhance the management and regulation level of municipal and district centers
System efficiency	Streamlined patient flow between prehospital and in-hospital care	Faster prehospital response and flexible decision-making	Integrated command centers and emergency coordination mechanisms

To evaluate the impact and sustainability of Beijing’s independent legal entity reform of district-level emergency medical centers, both performance and governance indicators should be systematically tracked. We propose a comprehensive monitoring framework focusing on service performance, financial sustainability, human resource stability, and management capacity, as shown in Table 3.

**Table 3:** Proposed framework for monitoring post-transition performance and governance indicators

Category	Key indicators	Description and policy relevance
Service capacity and performance	• Annual number of emergency dispatches	Quantitative measures to assess service efficiency, timeliness, and public satisfaction following the reform.
	• Average response time	
	• Emergency call fulfillment rate	
	• Service satisfaction rate	
Human resource stability	• Workforce turnover rate	Reflects personnel stability and professional development under the independent model.
	• Proportion of full-time prehospital staff	
	• Training participation rate	
Financial sustainability	• Government subsidy per dispatch	Evaluates financial viability and accountability of emergency centers operating autonomously.
	• Cost recovery rate	
	• Financial transparency audit score	
Governance and management capacity	• Existence of internal performance evaluation systems	Tracks the institutional and managerial improvements that are central to the reform’s objectives.
	• Data-sharing and coordination mechanisms with hospitals and local centers/stations	
	• Integration with district-level emergency command departments	
System resilience and responsiveness	• Frequency and effectiveness of multi-agency drills	Measures the ability of the system to respond effectively to large-scale or complex emergencies.
	• Capacity for on-site command during emergencies	
	• Real-time data reporting to municipal command center

Box 3 Dual roles of district-level emergency medical centers in Beijing District-level emergency medical centers in Beijing play a dual role within the city’s prehospital emergency medical service system. Professionally, they are guided by the Beijing Emergency Medical Center, which provides unified dispatching, technical oversight, and operational coordination. Administratively, however, they are managed by their respective district governments, which are responsible for human resource development, internal management, and the integration of prehospital emergency services into broader local emergency and health governance.

According to *Beijing Municipal Emergency Response Plan for Public Health Emergencies*, Beijing’s emergency response system operates under a four-tier framework, in which the level of response corresponds to the scale and severity of an incident. Level IV emergencies are handled primarily by district governments; Level III responses may be coordinated by either district or municipal governments depending on the specific situation; Level II responses fall under the authority of the municipal government; and Level I emergencies require reporting to the central government and are managed under national coordination. This system ensures that every level of government maintains a defined scope of decision-making and operational responsibility.

Within this framework, district-level emergency medical centers serve as the key operational bodies for prehospital emergency management within their jurisdictions. During Level IV, they are responsible for on-site command of prehospital emergency medical service, coordination with district emergency authorities, and management of rescue operations and patient transfers. In higher-level emergencies, district-level centers may also temporarily assume command responsibilities on behalf of the BEMC until municipal-level personnel arrive on site, at which point authority is formally transferred.

This hierarchical yet flexible structure reflects a defining characteristic of China’s administrative and emergency management systems. It ensures that both vertical coordination and local responsiveness are maintained. District-level centers thus act as an indispensable link—bridging municipal command with frontline implementation—and play a vital role in ensuring that Beijing’s prehospital EMS system remains efficient, responsive, and resilient.

## Future outlook

Beijing’s independent legal entity reform of its major district-level emergency medical centers offers valuable insights and lessons for other regions in China. In economically developed cities, where emergency demands are high and resources more abundant, this model can significantly enhance the professionalism and operational efficiency of emergency services. The independence allows for better financial management, streamlined decision-making, and a focus on specialized prehospital care. These advantages can lead to improved service quality, faster response times, and greater accountability in resource allocation.

However, in less-developed regions with lower emergency demand and limited government subsidies, the independent legal entity model may face greater operational difficulties, such as underfunding and workforce shortages. For instance, in Zhangjiakou—a smaller, less-developed secondary city adjacent to Beijing—all prehospital emergency medical centers still operate under the affiliation model. Most of these centers do not have independent or dedicated ambulance teams. The primary constraints are insufficient financial resources, limited human capital, and a lack of administrative positions to support independent institutional establishment. These challenges highlight that while Beijing’s model can be successful in specific contexts, it is not a one-size-fits-all solution. Tailoring the reform to the local context is essential, and directly replicating Beijing’s model across different regions may not be practical or effective.

Beijing’s reform, it should be noted, was implemented at the district level and should not be understood simply as a reform of individual institutions. Rather, it represents a system-oriented initiative to strengthen overall emergency management capacity across the city. For a megacity such as Beijing, establishing district-level emergency sub-centers has reinforced the concept of regionalized management, enabling each district to coordinate and oversee prehospital emergency operations within its jurisdiction. This approach allows districts to tailor their management priorities, workforce training, and operational strategies based on their distinct geographic, demographic, and functional characteristics. By doing so, the system achieves layered and differentiated governance, which helps address the inherent challenges of managing an extensive and complex urban EMS network. Overall, the reform marks a transition from institutional restructuring to systemic enhancement, emphasizing governance connectivity, efficiency, and resilience within Beijing’s prehospital EMS framework.

At the local level, however, whether further independent reform of emergency centers/stations is necessary remains open to discussion. The prevailing view in Beijing’s policy community is to respect local conditions and maintain flexibility. In remote or mountainous districts, local centers/stations must remain affiliated with nearby medical institutions due to infrastructure constraints and resource limitations. In these areas, affiliation model remains the most practical arrangement for ensuring equipment maintenance, staffing, and service continuity. Therefore, future reform efforts should prioritize differentiated strategies based on geography, capacity, and resource availability, ensuring that system integration and local feasibility advance in parallel.

To fully realize the potential of this model, future reforms should prioritize key areas such as improving financial support, refining fee structures, and addressing human resource challenges. Additionally, enhancing training and continuous professional development opportunities for emergency medical teams will be crucial for maintaining high standards of care and ensuring resilience in the face of increasing demand. By focusing on these areas, the reform can support the long-term sustainability of emergency medical services, ensuring that they are capable of adapting to the evolving needs of modern cities while maintaining a high level of quality and safety.

Ultimately, a balanced approach that integrates both management and operational flexibility will be essential for preparing emergency services to meet future challenges. By addressing the financial, workforce, and training needs of emergency centers, the reform can contribute to a more resilient and capable emergency medical service system, better equipped to safeguard public health and safety in the long run.

## Data Availability

Data sharing not applicable as no datasets generatedand/or analyzed for this study.
